# Supplementary material for: Polycomb repressive complexes 1 and 2 independently and dynamically regulate euchromatin during cerebellar neurodevelopment
Source: PLoS Genet. 2025 Sep 29;21(9):e1011843. doi: 10.1371/journal.pgen.1011843 (PMC12500107; doi:10.1371/journal.pgen.1011843)
Supplement: S1 Table — (DOCX) [file pgen.1011843.s001.docx]

**S1 Table: Reagents and other tools used in this study**

| REAGENT or RESOURCE | SOURCE | IDENTIFIER |
| --- | --- | --- |
| **Antibodies** | | |
| H2AK119ub | CST | CAT# 8240S  RRID: AB_10891618 |
| H3K4me1 | CST | CAT#5326T RRID: AB_10695148 |
| H3K27me3 | CST | CAT# 9733S  RRID: AB_2616029 |
| H3K9me3 | Active Motif | CAT# 39065  RRID: AB_2793334 |
| H3K27ac | Active Motif | CAT# 39133  RRID: AB_2561016 |
| H3K4me3 | CST | CAT# 9751S  RRID: AB_2616028 |
| H3K27me1 | Active Motif | CAT# 61016  RRID: AB_2715573 |
| H3K9ac | Active Motif | CAT# 39138  RRID: AB_2561017 |
| **Chemicals, Peptides, and Recombinant Proteins** | | |
| Nuclei EZ Lysis Buffer | Sigma-Aldrich | CAT# N3408-200ML |
| Proteas and Phosphatase inhibitor cocktail | Thermo-Science | CAT# 1891280 |
| Sodium Butyrate | Sigma-Aldrich | CAT# 19-137 |
| K-MetStat Panel | Epicypher | CAT# 19-1002 |
| DMEM/F-12, HEPES | Life Technologies | CAT# 11330032 |
| 10% Fetal Bovine Serum | Life Technologies – Invitrogen | CAT# A5256701 |
| PFA Ampules | Thermo-Fisher | CAT# 28906 |
| Spermidine | Sigma-Aldrich | CAT# 85558-1G |
| Complete Mini, EDTA-free | Roche | CAT# 11836170001 |
| Iodoacetamide | Millipore-Sigma | CAT# A3221-1VL |
| Triton X-100 | Fisher Bioreagents | CAT# BP151-500 |
| Digitonin | Sigma-Aldrich | CAT# D141 |
| pAG-MNase | Epicypher | CAT# 15-1016 |
| **Deposited Data** | | |
| Raw data and analyzed data | This paper | NCBI BioProject PRJNA1150596 |
| **Experimental Models: Organisms/Strains** | | |
| Wild-type Jackson C57BL/6 | The Jackson Laboratory | Cat# 000664 |
| **Software and Algorithms** | | |
| DiffBind | ^63,64^ |  |
| SEACR | ^54^ |  |
| MACS2 | ^55^ |  |
| SAMtools v.1.14 | ^113^ |  |
| BEDTools | ^116^ |  |
| ShinyGO | ^110^ |  |
| ChromHMM | ^75^ |  |
| KaryoplotR v.1.22.0 | ^114^ |  |
| FastQC |  | https://www.bioinformatics.babraham.ac.uk/projects/fastqc/ |
| deepTools | ^62^ |  |
| DESeq2 | ^109^ |  |
| GraphPad Prism10 |  |  |
| Adobe Illustrator 2021 |  |  |
| **Other** |  |  |
| CUT & RUN kit | Epicypher | CAT# 14-0050 |
| Concanavalin A-coated magnetic beads | CUTANA | CAT# 21-1401 |
| Rneasy Mini Kit | Qiagen | CAT# 74104 |
| NEBNext Ultra II DNA Library Prep Kit | New England Biolabs | CAT# E7645L |
