# Supplementary figures and images for: Polycomb repressive complexes 1 and 2 independently and dynamically regulate euchromatin during cerebellar neurodevelopment
Source: PLoS Genet. 2025 Sep 29;21(9):e1011843. doi: 10.1371/journal.pgen.1011843 (PMC12500107; doi:10.1371/journal.pgen.1011843)

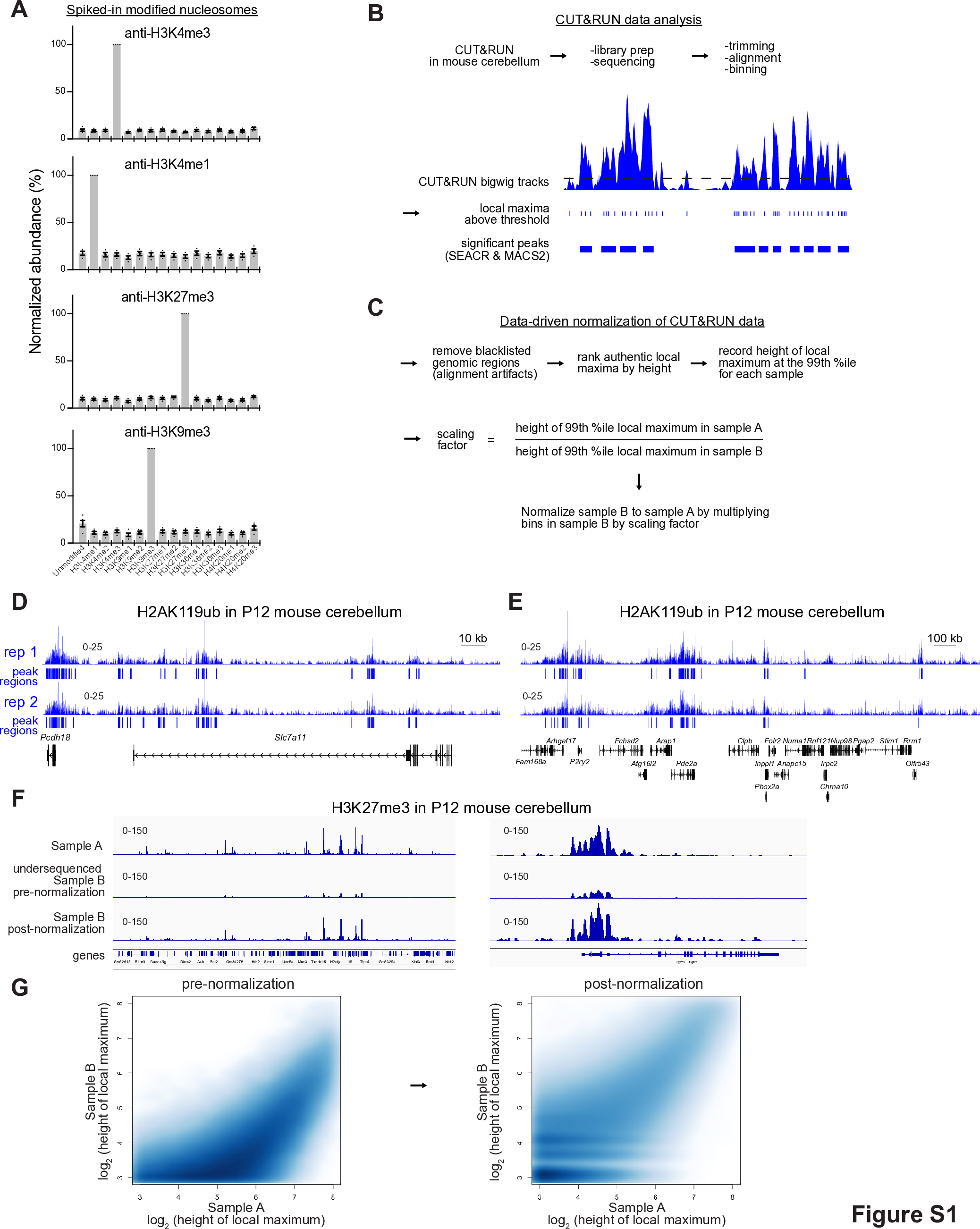

Supplement: S1 Fig — (A) A panel of nucleosomes modified by specific histone PTMs and harboring modification-specific DNA sequences were spiked-into CUT&RUN experiments in mouse cerebellum using antibodies to H3K4me3, H3K4me1, H3K27me3, and H3K9me3. Nucleosomes modified by acetylation were not present in this panel. The number of reads derived from DNA sequences associated with each spiked-in modified nucleosome was normalized to the total number of reads derived from all the spiked-in modified histones. (B) Overview of pipeline for analyzing CUT&RUN data. (C) Strategy for normalizing CUT&RUN data. (D) Normalized CUT&RUN data for H2AK119ub in two biological replicates. Peaks called using SEACR. (E) Normalized H2AK119ub data across a broader genomic region. (F) Coverage tracks of CUT&RUN data for H3K27me3 detected in the mouse cerebellum, followed by normalization of shallowly sequenced sample B to deeply sequenced sample A. (G) Scatter plot comparison of peak heights in H3K27me3 CUT&RUN data from mouse cerebellum between samples A and B before and after normalization. (TIF) [file pgen.1011843.s004.tif]

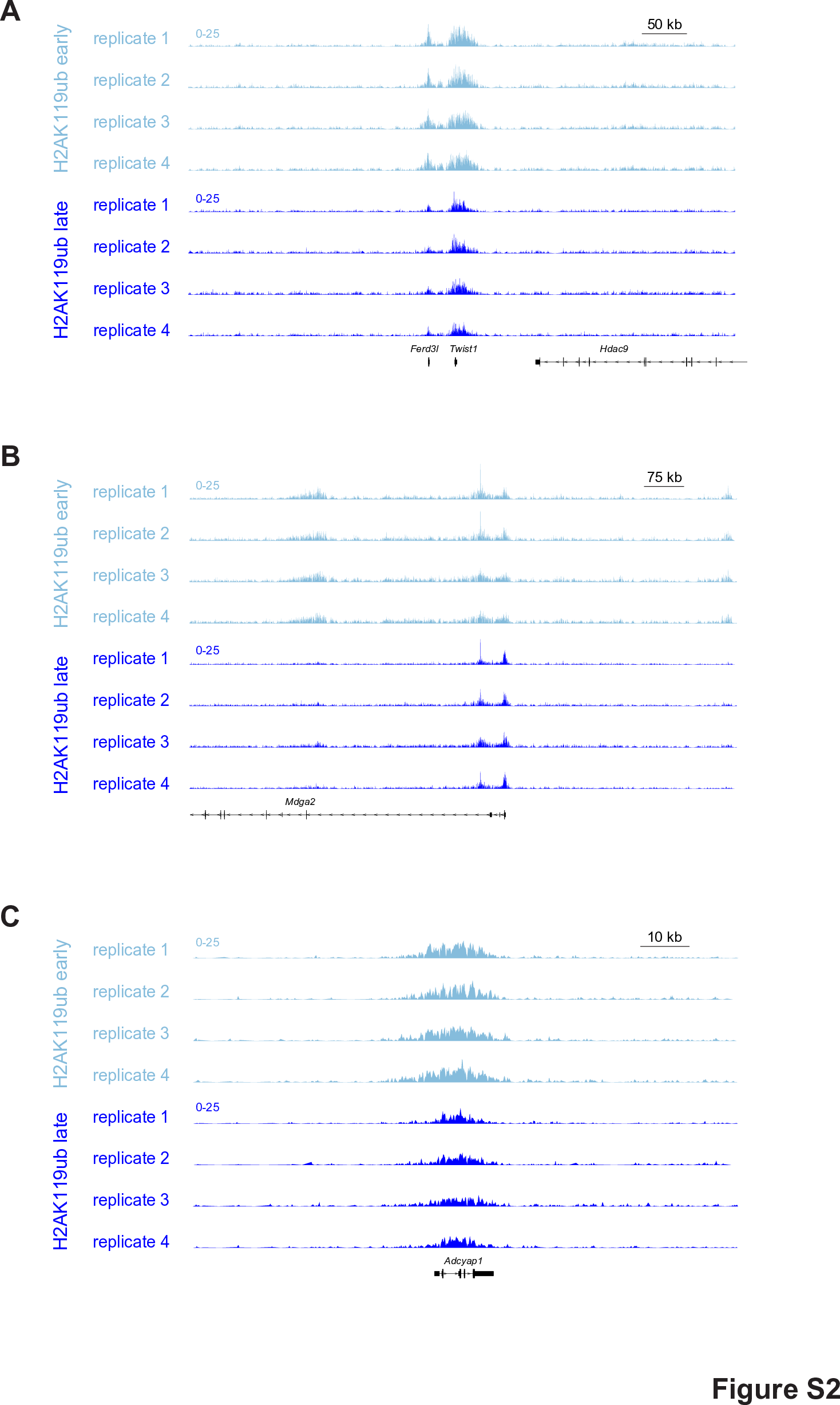

Supplement: S2 Fig — (A-C) Genome browser tracks showing normalized H2AK119ub CUT&RUN signal across four biological replicates from early (P12) and late (3 month) mouse cerebellum. (TIF) [file pgen.1011843.s005.tif]

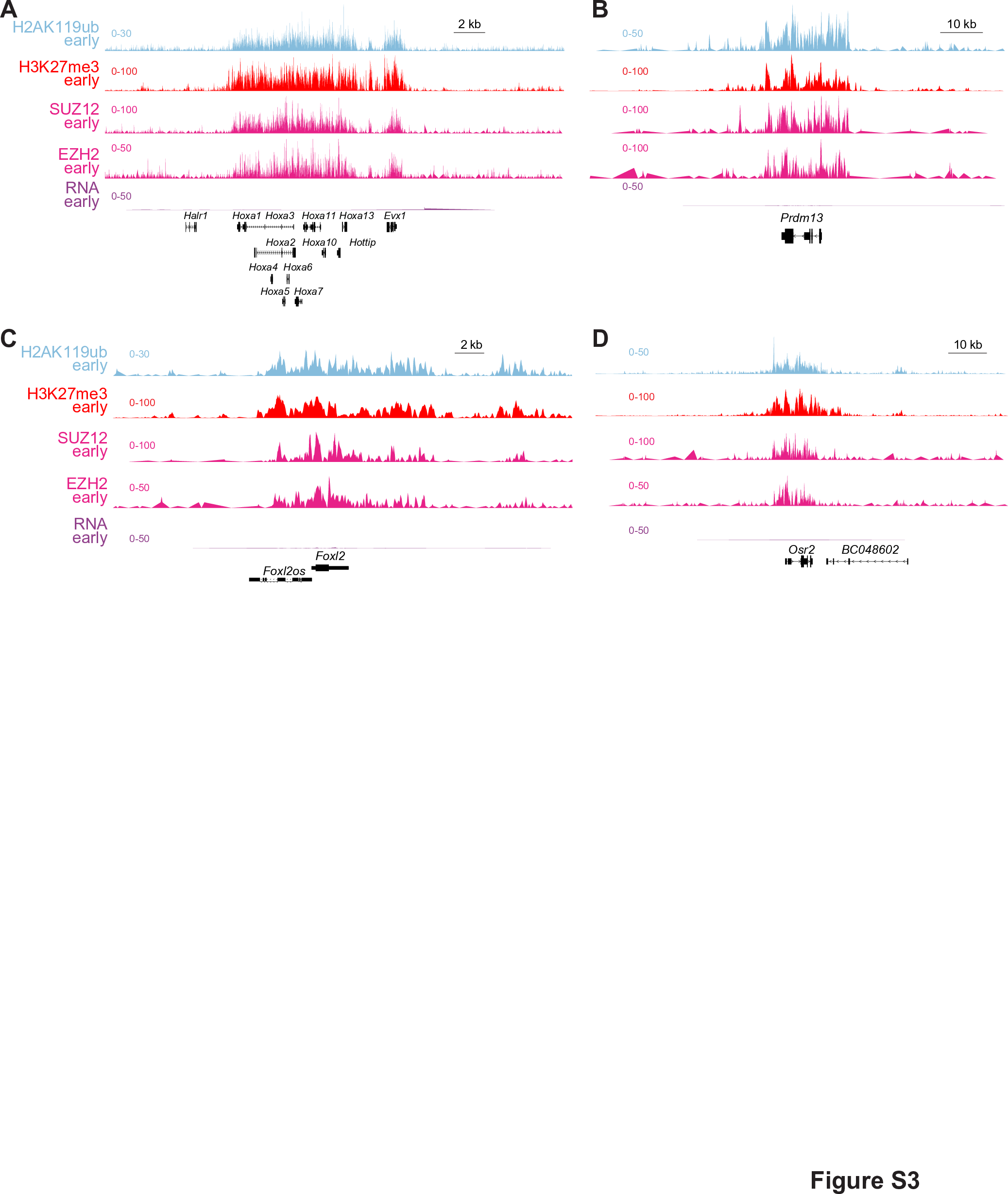

Supplement: S3 Fig — (A-D) CUT&RUN detection of H2AK119ub, H3K27me3, EZH2, and SUZ12 in the early cerebellum at repressed loci encoding transcription factors. (TIF) [file pgen.1011843.s006.tif]

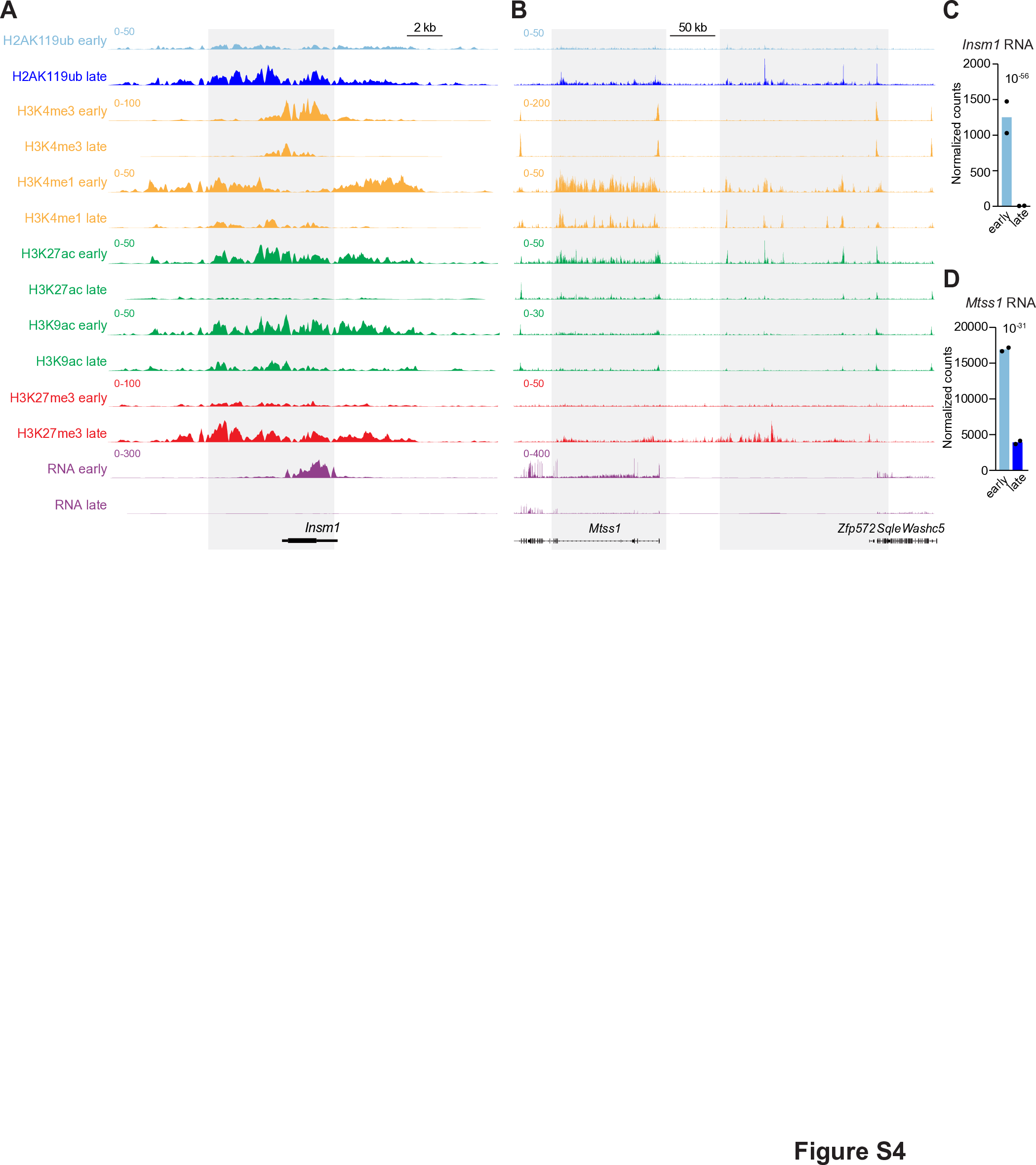

Supplement: S4 Fig — (A) CUT&RUN detection of the indicated histone modifications at the Insm1 locus in early and late cerebellum shown alongside RNAseq tracks. (B) CUT&RUN and RNAseq tracks at the Mtss1 locus. (C) Normalized counts for RNAseq detection of Insm1 transcripts in nuclei isolated from early and late cerebellum (n = 2, adjusted p-value computed by DESeq2). (D) Same as in (C), for Mtss1. (TIF) [file pgen.1011843.s007.tif]

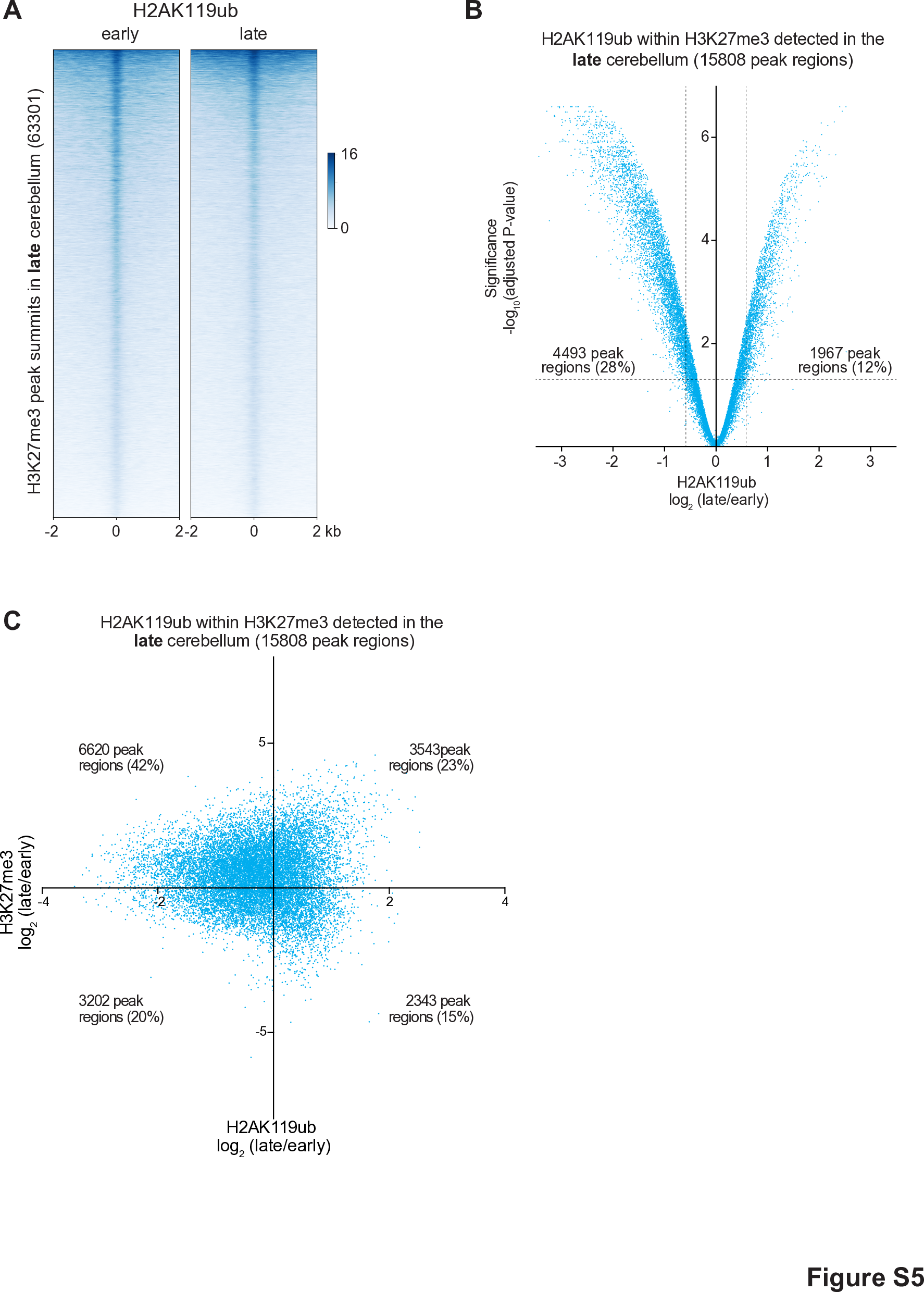

Supplement: S5 Fig — (A) Heatmaps depicting normalized H2AK119ub CUT&RUN data in the early and late cerebellum centered around peak summits for H3K27me3 detected in the late cerebellum. (B) Volcano plot depicting H2AK119ub abundance, as detected in normalized CUT&RUN data from early and late cerebellum, within H3K27me3 peak regions detected in the late cerebellum. The significance threshold was an adjusted p-value of <0.05, as computed by edgeR and Limma (n = 4). (C) Log2-log2 scatterplot comparing the ratio of H2AK119ub (late/early) to the ratio of H3K27me3 (late/early) within H3K27me3 peak regions identified in the early cerebellum (n = 4 for H2AK119ub, n = 2 for H3K27me3). (TIF) [file pgen.1011843.s008.tif]

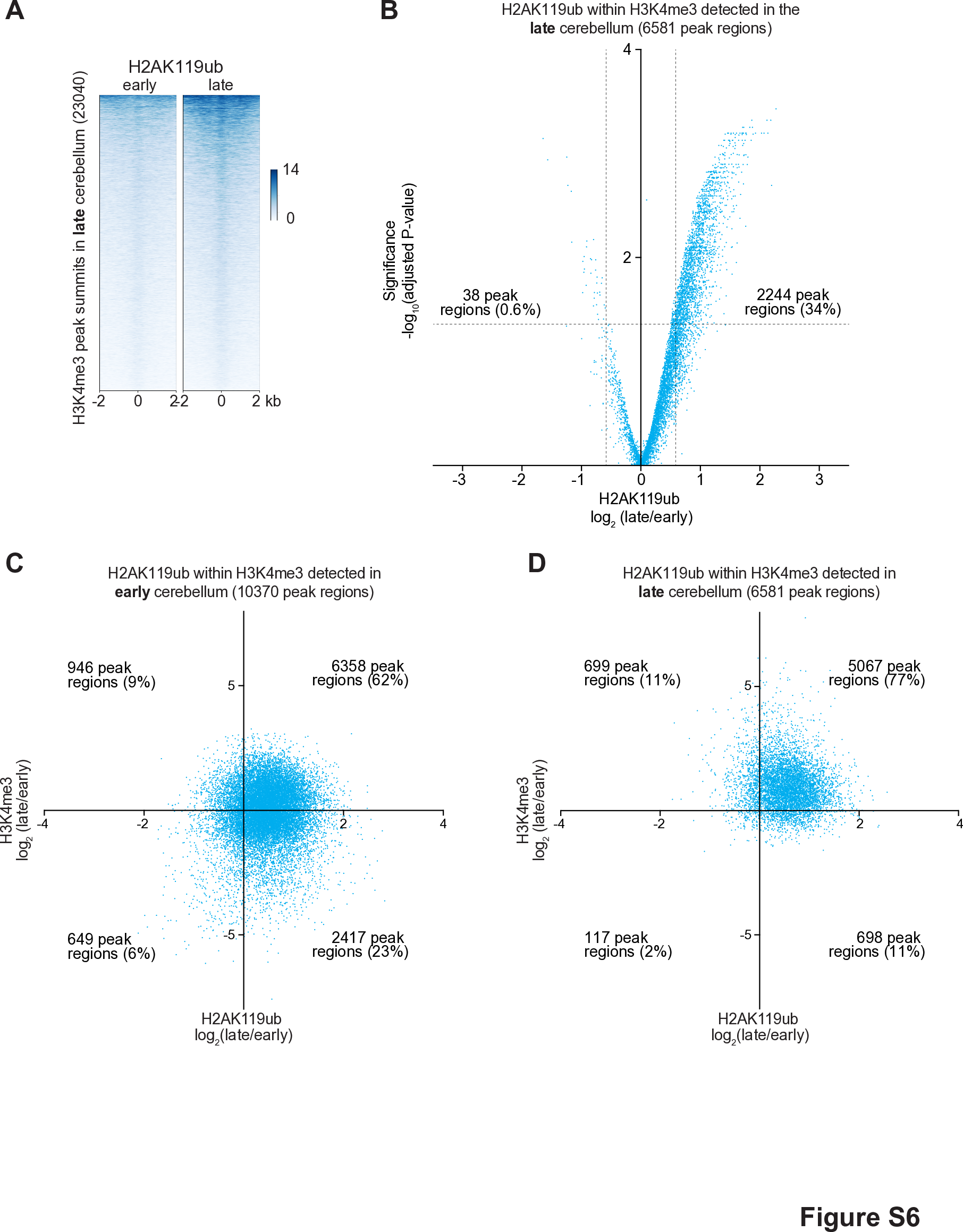

Supplement: S6 Fig — (A) Heatmaps depicting normalized H2AK119ub CUT&RUN data in the early and late cerebellum centered around peak summits identified by MACS2 narrow for H3K4me3 in the late cerebellum. (B) Volcano plot depicting H2AK119ub abundance, as detected in normalized CUT&RUN data from early and late cerebellum, within H3K4me3 peak regions detected in the late cerebellum. The significance threshold was an adjusted p-value of <0.05, as computed by edgeR and Limma (n = 4). (C) Log2-log2 scatterplot comparing the ratio of H2AK119ub (late/early) to the ratio of H3K4me3 (late/early) within H3K4me3 peak regions identified in the early cerebellum (n = 4 for H2AK119ub, n = 2 for H3K4me3). (D) Log2-log2 scatterplot comparing the ratio of H2AK119ub (late/early) to the ratio of H3K4me3 (late/early) within H3K4me3 peak regions identified in the late cerebellum (n = 4 for H2AK119ub, n = 2 for H3K4me3). (TIF) [file pgen.1011843.s009.tif]

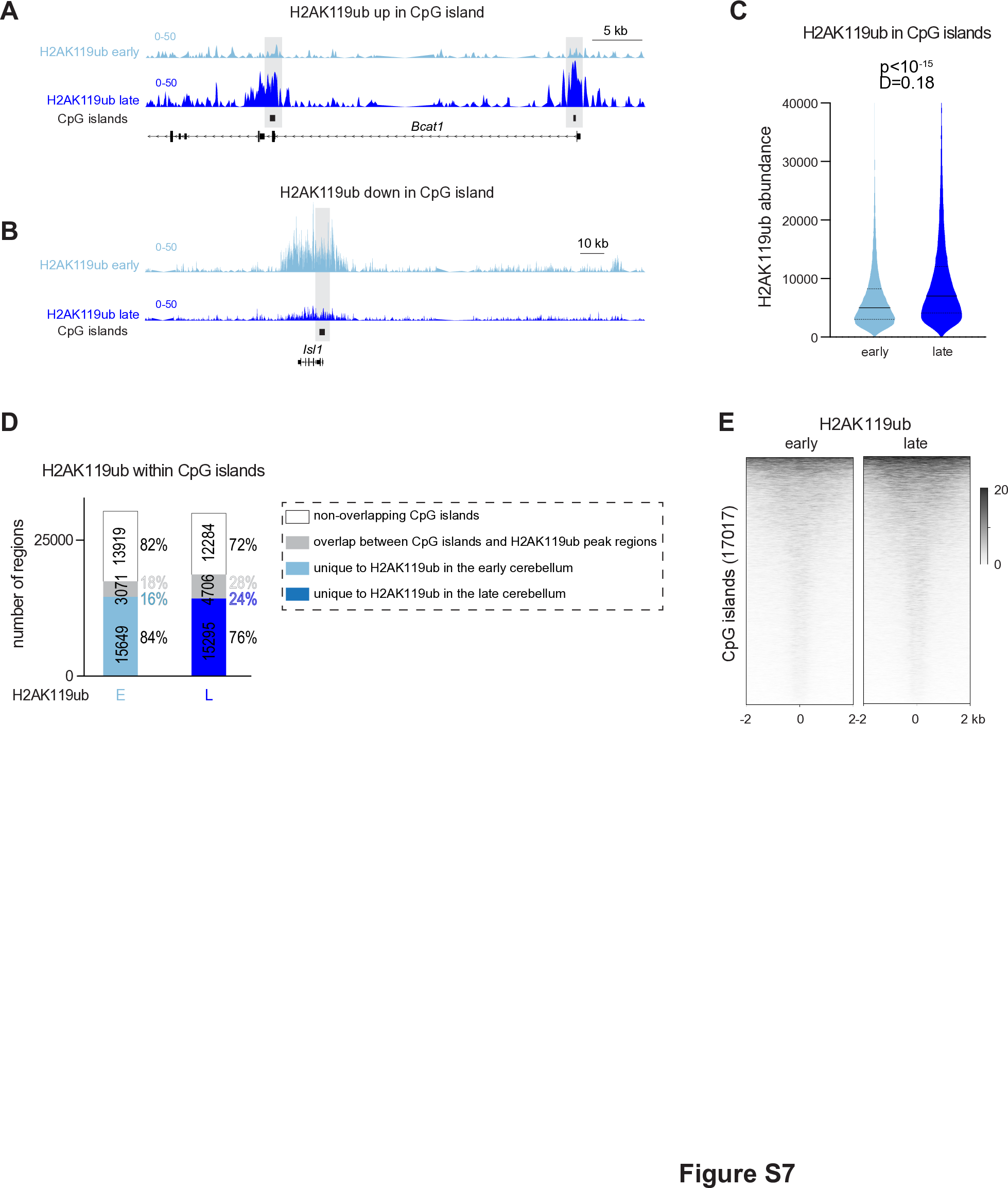

Supplement: S7 Fig — (A) CUT&RUN detection of H2AK119ub in the cerebellum within the Bcat1 locus harboring promoter-associated CpG islands. Shaded regions highlight CpG islands exhibiting neurodevelopmental increase in H2AK119ub. (B) Same as (A), showing neurodevelopmental decrease in H2AK119ub at the Isl1 locus. (L) Violin plots depicting the length-scaled abundance of H2AK119ub in the early and late cerebellum within CpG islands. P-value and D statistic by K-S D test (n = 4). (C) Fraction of H2AK119ub peak regions that overlap with CpG islands in the early and late cerebellum. The two middle percentages reflect: the fraction of CpG islands that overlap with H2AK119ub peak regions and the fraction of H2AK119ub peak regions that overlap with CpG islands. (D) Heatmaps depicting normalized H2AK119ub CUT&RUN data in the early and late cerebellum centered around CpG islands. (TIF) [file pgen.1011843.s010.tif]

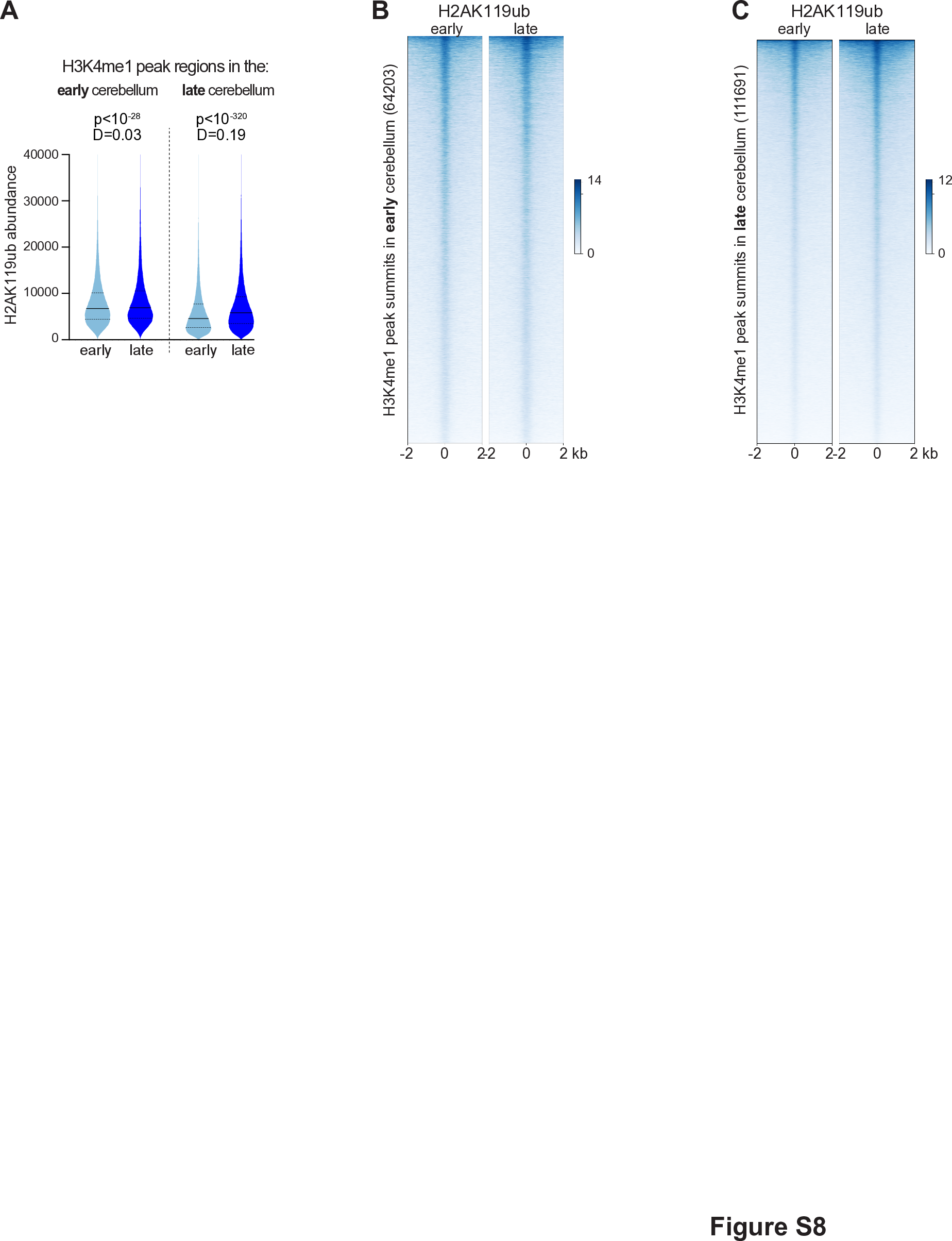

Supplement: S8 Fig — (A) Violin plots showing length-scaled H2AK119ub abundance within H3K4me1 peak regions detected in the early and late cerebellum. P-value and D statistic by K-S D test (n = 4). (B) Heatmaps depicting normalized H2AK119ub CUT&RUN data in the early and late cerebellum centered around H3K4me1 peak summits identified by the MACS2 narrow algorithm in the early cerebellum. (C) Same as (B), shown for peak summits detected in the late cerebellum. (TIF) [file pgen.1011843.s011.tif]

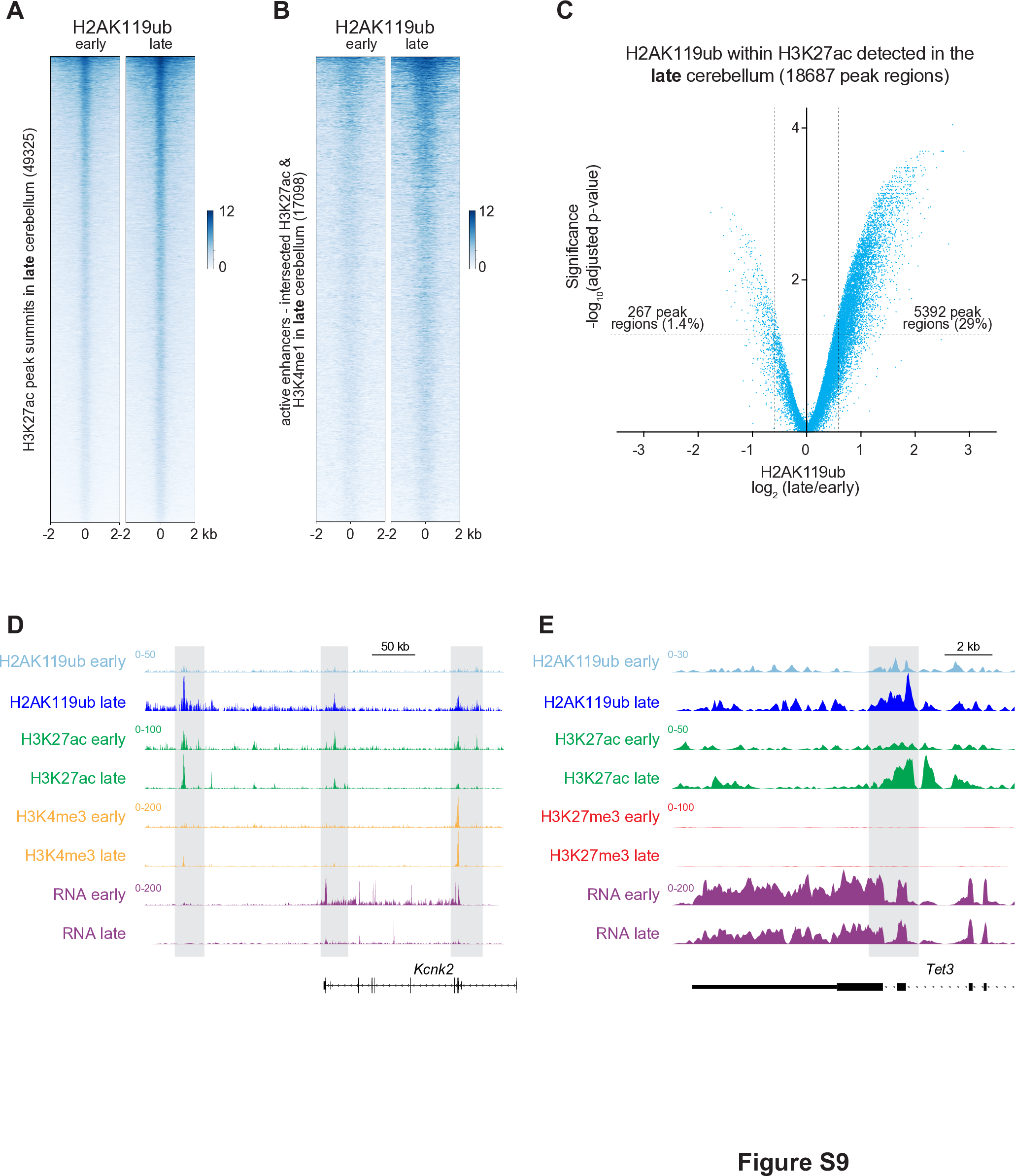

Supplement: S9 Fig — (A) Heatmaps depicting normalized H2AK119ub CUT&RUN data centered around H3K27ac peak summits identified by MACS2 narrow in the late cerebellum. (B) Heatmaps depicting normalized H2AK119ub CUT&RUN data centered around active enhancers (intersection of SEACR-defined H3K27ac peaks and MACS2 broad H3K4me1 peaks) detected in the late cerebellum. (C) Volcano plot depicting H2AK119ub abundance, as detected in normalized CUT&RUN data from early and late cerebellum, within H3K27ac peak regions detected in the late cerebellum. The significance threshold was an adjusted p-value of <0.05, as computed by edgeR and (n = 4). (D) CUT&RUN and RNAseq tracks showing an active enhancer cluster with neurodevelopmental gain of H2AK119ub. (E) CUT&RUN and RNAseq tracks showing an active enhancer cluster with neurodevelopmental gain of H2AK119ub and H3K27ac. (TIF) [file pgen.1011843.s012.tif]

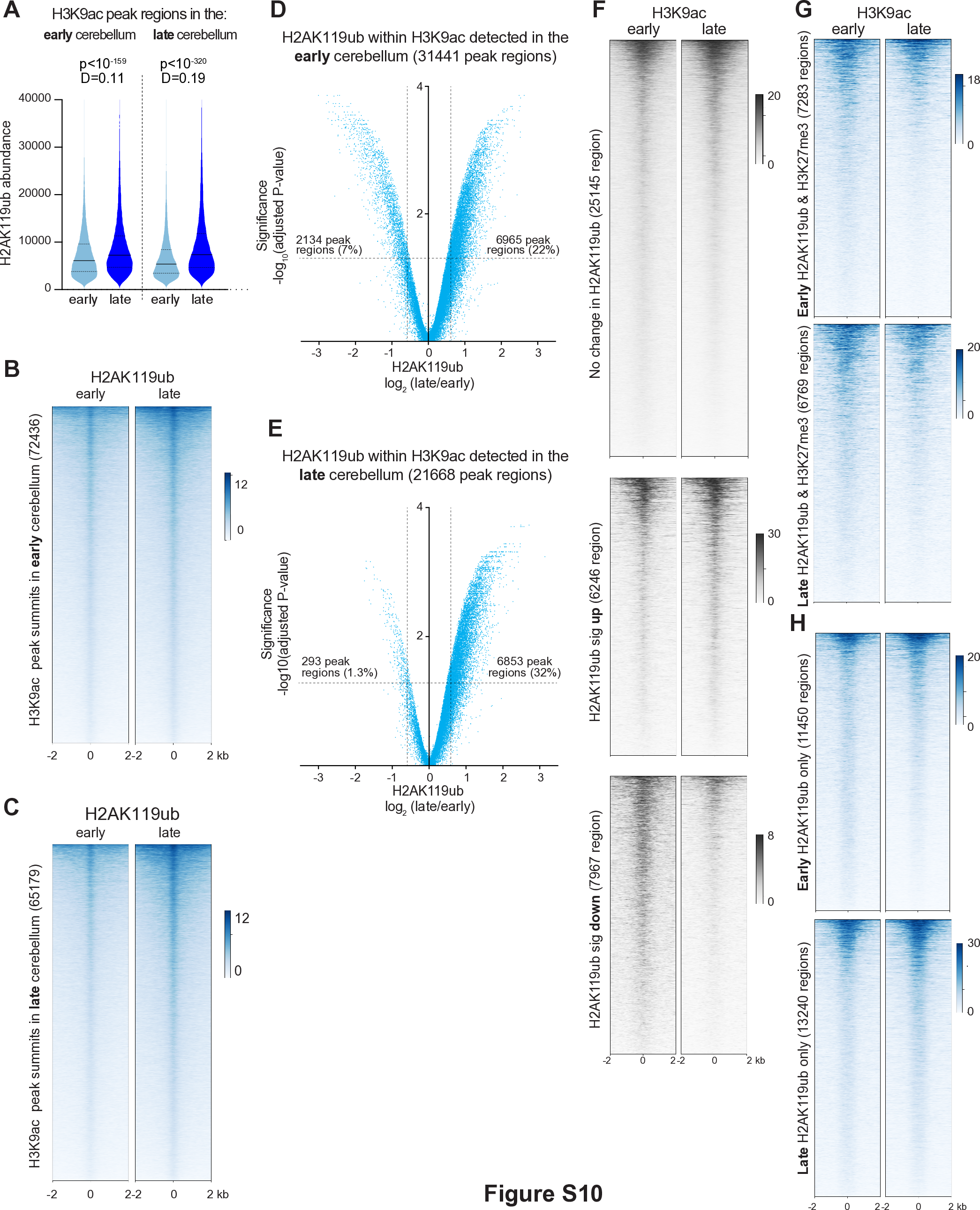

Supplement: S10 Fig — (A) Violin plots showing length-scaled H2AK119ub abundance within H3K9ac peak regions detected in the early and late cerebellum. P-value and D statistic by K-S test (n = 4). (B) Heatmaps depicting H2AK119ub CUT&RUN data centered on MACS narrow-defined H3K9ac peak summits from early cerebellum. (C) Heatmaps depicting H2AK119ub CUT&RUN data centered on H3K9ac peak summits from late cerebellum. (D) Volcano plot depicting H2AK119ub abundance, as detected in normalized CUT&RUN data from early and late cerebellum, within H3K27ac peak regions detected in the early cerebellum. The significance threshold was an adjusted p-value of <0.05, as computed by edgeR and Limma (n = 4). (E) Volcano plot depicting H2AK119ub abundance, as detected in normalized CUT&RUN data from early and late cerebellum, within H3K27ac peak regions detected in the late cerebellum. The significance threshold was an adjusted p-value of <0.05, as computed by edgeR and Limma (n = 4). (F) Heatmaps depicting H3K9ac CUT&RUN data within loci with no change, increased or decreased H2AK119ub over neurodevelopment. (G) Heatmaps depicting H3K9ac CUT&RUN data within loci harboring both H2AK119ub and H3K27me3. (H) Heatmaps depicting H3K9ac CUT&RUN data within loci harboring H2AK119ub in the absence of H3K27me3. (TIF) [file pgen.1011843.s013.tif]

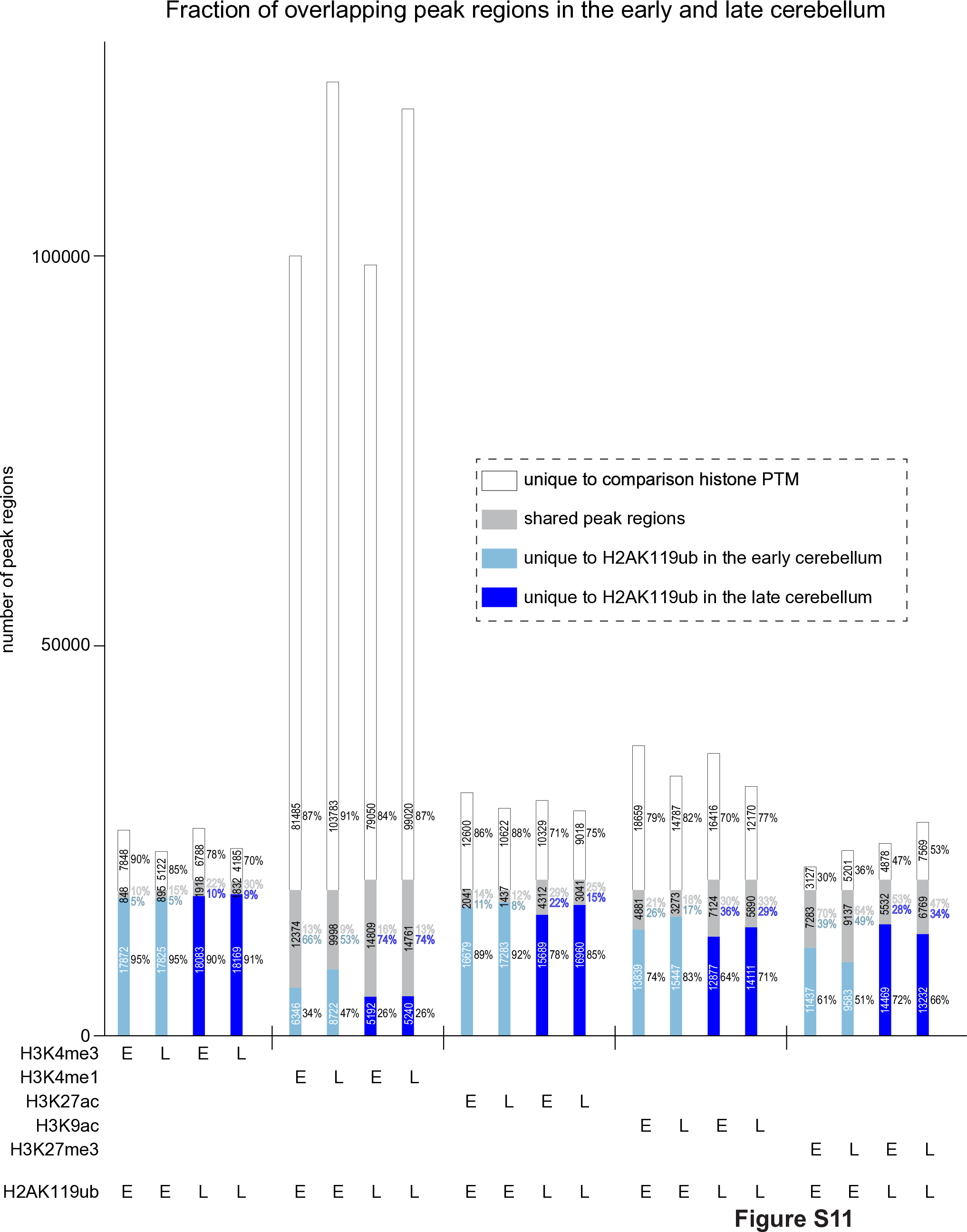

Supplement: S11 Fig — Fraction of SEACR-defined H2AK119ub peak regions that overlap with peak regions for different histone modifications detected in the early and late cerebellum. Peak regions were called using the SEACR algorithm for all modifications except H3K4me1, when MACS2 broad was used. Inputted peak regions reflect the consensus between two replicates, defined by intersecting overlapping regions found in both. Percentages at the top reflect the fraction of peak regions for each modification that overlap with H2AK119ub peak regions. Bottom percentages reflect the fraction of H2AK119ub peak regions that overlap with peak regions for the comparison modification. (TIF) [file pgen.1011843.s014.tif]

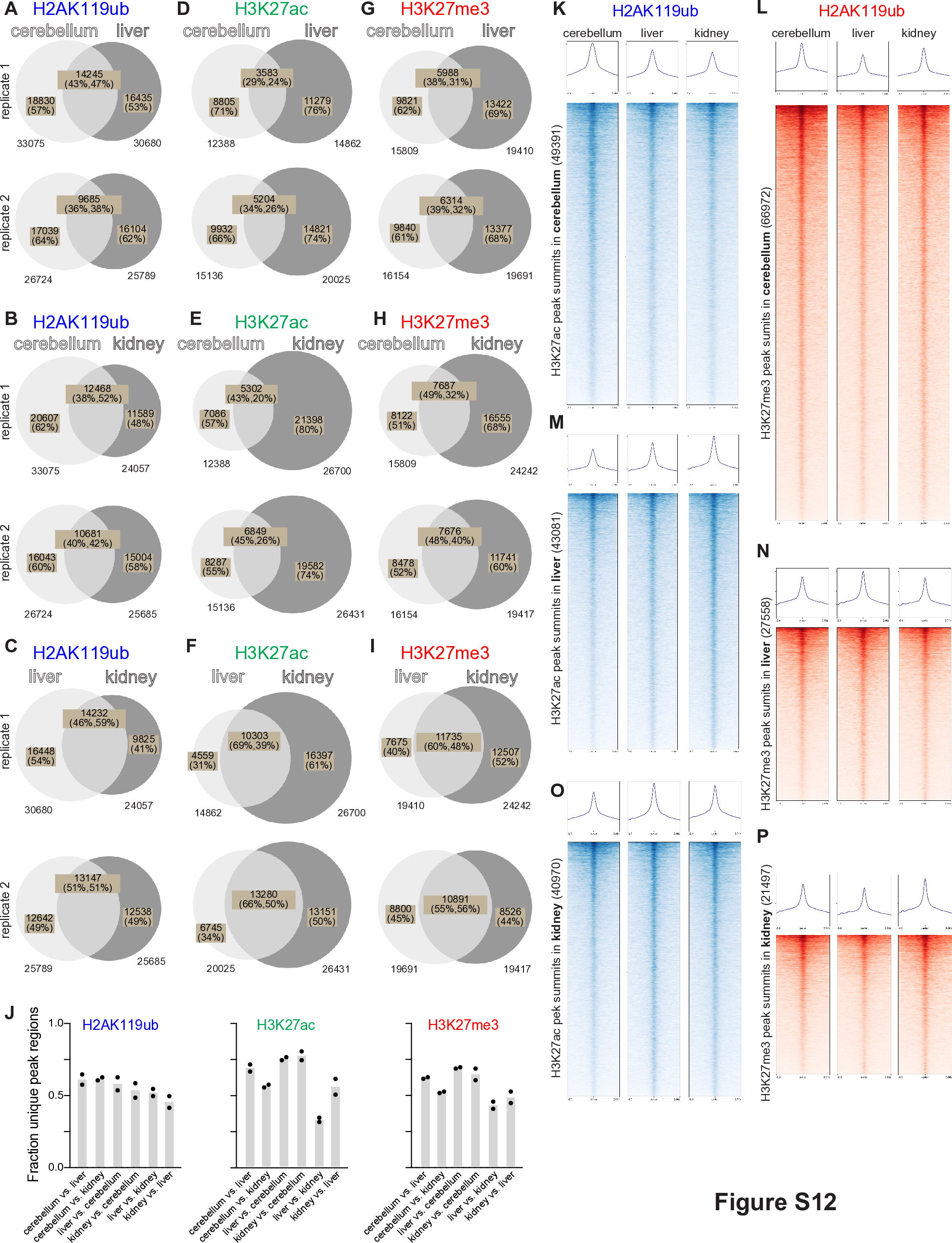

Supplement: S12 Fig — (A-C) Venn diagrams depicting the overlap between SEACR-called peak regions for H2AK119ub CUT&RUN in the cerebellum and liver (A), cerebellum and kidney (B), and liver and kidney (C). (D-F) Same as (A-C), for H3K27ac. (G-I) Same as (A-C), for H3K27me3. (J) Bar graphs depicting the fraction of unique peak regions. (K–P) Heatmaps of H2AK119ub in cerebellum, liver, and kidney centered on H3K27ac or H3K27me3 peak summits called in cerebellum (K–L), liver (M–N), or kidney (O–P). (TIF) [file pgen.1011843.s015.tif]

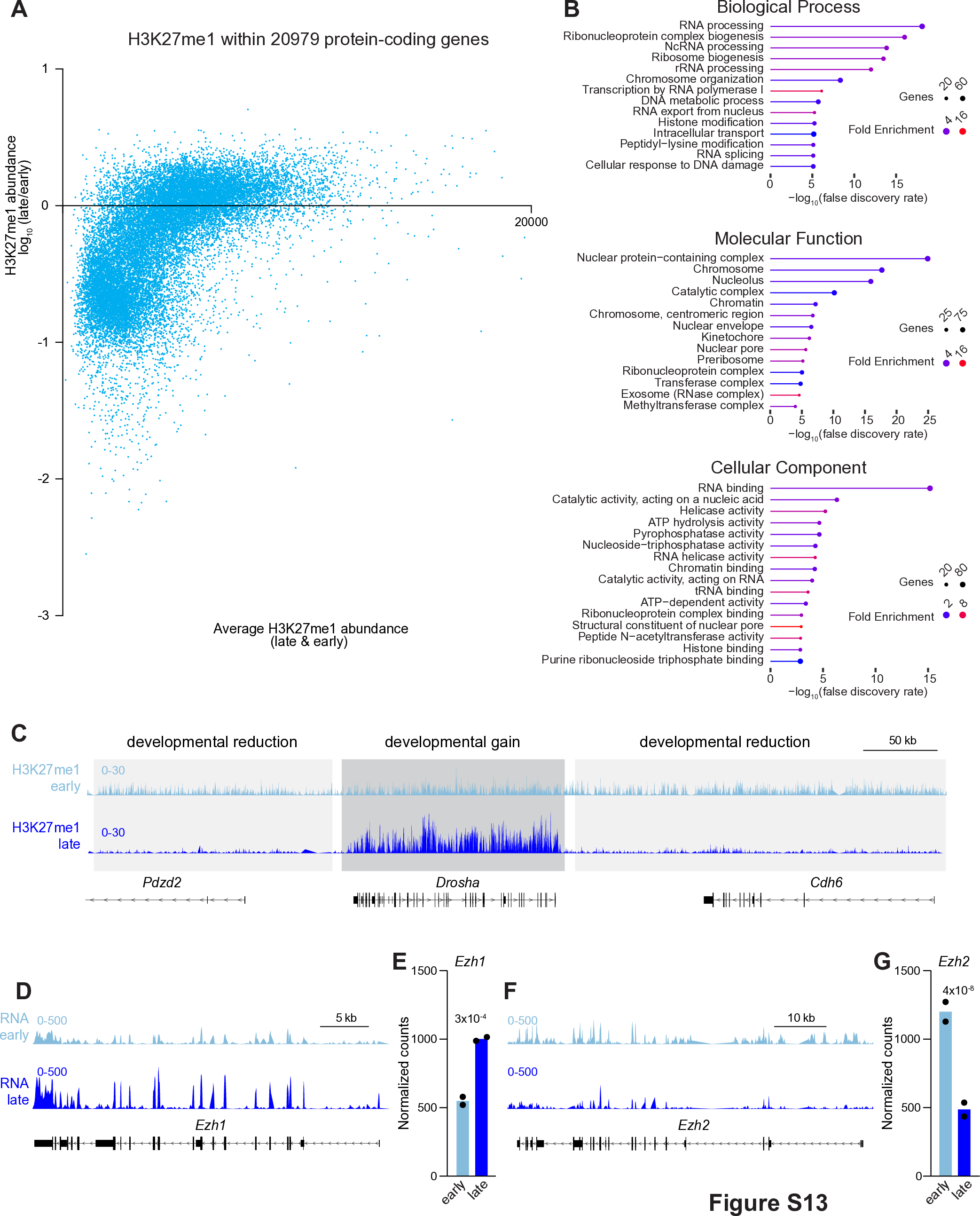

Supplement: S13 Fig — (A) Minus-average plot comparing the length-scaled bundance of H3K27me1 across all protein-coding genes. (B) ShinyGO analysis of 526 genes exhibiting > 2-fold increase in H3K27me1 over neurodevelopment. (C) H3K27me1 CUT&RUN tracks at the Drosha locus. (D) RNAseq tracks at the Ezh1 locus in early and late cerebellum. (E) Bar graph comparing DESeq2 normalized Ezh1 mRNA counts in early and late cerebellum. (F) Same as (D), at the Ezh2 locus. (G) Same as (E), at the Ezh2 locus. (TIF) [file pgen.1011843.s016.tif]
