# Supplementary material for: Polycomb repressive complexes 1 and 2 independently and dynamically regulate euchromatin during cerebellar neurodevelopment
Source: PLoS Genet. 2025 Sep 29;21(9):e1011843. doi: 10.1371/journal.pgen.1011843 (PMC12500107; doi:10.1371/journal.pgen.1011843)
Supplement: Attachment — Submitted filename: rebuttal.docx [file pgen.1011843.s018.docx]

We deeply appreciate the time and thought the reviewers put into their comments. In our efforts to address the questions they raised, we have gained new insights into our own data since the original submission and strengthened/clarified the message in the revised manuscript. Below, we quote the original reviewer comments in *italics* and respond in roman font.

**Reviewer 1**

*This manuscript presents a thorough analysis of the developmental dynamics of H2AK119 ubiquitination (K119ub) and H3K27 monomethylation (K27me1) in relation to both active and repressive chromatin marks. While the study is primarily descriptive, it offers novel insights into the potential functions of two underexplored chromatin modifications, particularly within a neurodevelopmental framework. The authors take advantage of the cellular homogeneity provided by the predominance of granule neurons in the developing (P12) and mature (3-month-old) cerebellum to investigate the genome-wide distribution of K119ub and K27me1. Using Cut&Run, they analyze these marks alongside enhancer-, promoter-, and heterochromatin-associated histone modifications. Notably, the authors creatively integrate a range of computational and statistical approaches to enhance the interpretation of these complex datasets. Their analyses reveal that the association of K119ub and K27me1 with active and repressive chromatin regions is both context- and developmental stage–dependent, and substantially more dynamic than previously suggested by studies focused on the canonical PRC1/2 complexes and their associated mark, H3K27me3. In particular, K119ub is enriched in regions that lose active promoter and enhancer marks during cerebellar maturation, whereas K27me1 is broadly distributed across euchromatic regions with no clear association with transcriptional activation or repression, nor predictive value for future transcriptional states.*

*The findings are significant, not only advancing our understanding of chromatin regulation in neural development, but also introducing computational and statistical methodological innovations that will likely be valuable for future studies—especially those involving dynamic, multifactorial chromatin landscapes.*

*While the integration of transcriptomic data could be more fully developed, I acknowledge the inherent challenges in aligning transcriptional outputs with chromatin dynamics, due to differences in sensitivity and variability. Therefore, I offer only minor suggestions aimed at improving the clarity of the manuscript and interpretation of certain figures.*

We sincerely thank the Reviewer for their appreciation of our study, its significance and the value of our methodological innovations to the field. We took the reviewer's point here to heart and have attempted to better integrate transcriptomic data in relation to the role of H2AK119ub at promoters, which is much more feasible than investigating the effects of H2AK119ub at enhancers. We have also strengthened our comparisons between H3K27me1 and RNAseq data.

*Minor Comments:*

*1-The change on K119ub abundance from early to late cerebellum on each of the early and late eu- or heterochromatin marks is very similar (within each mark). Is this just a coincidence or due to similar peaks identified for each of the marks in early and late cerebellum (i.e. how different/similar are the H3K27me3 peaks between early and late cerebellum)?*

The early and late peak regions are quite similar for all the histone modifications we have compared to H2AK119ub. In the revised manuscript, we now include panels in **Figure 6** showing the degree to which the early and late peak regions overlap for all the H3 modifications tested (K4me3, K4me1, K27ac, K9ac and K27me3). This new analysis shows that the fraction of overlapping peak regions is lower for H2AK119ub than for the other modifications, underscoring the fact that K119ub is the most dynamic of the marks tested.

*2-What is the transcriptional status of the bivalent promoters with increased abundance of K119ub from early to late cerebellum? Does this differ from the promoters that only have H3K4me3?*

These were excellent questions, and the additional analysis we performed to answer them (new **Figure 4H-M**) ended up adding something quite substantive to the manuscript. For both classes of promoters, those that undergo a significant increase in K119ub exhibit reduction of the downstream mRNA. This effect is not seen for genes whose promoters experience no change in K119ub. This observation strengthens our claim that K119ub is repressive, even when it appears at expressed loci.

*3-While H3K27ac is associated with active enhancers, there are instances of H3K27ac in poised or inactive enhancers as well. How certain are the authors that the increase of K119ub from early to late cerebellum in clusters of H3K27ac enhancer regions (Fig 4E-F) is associated with transcriptionally active genes? These figures would also benefit from showing the IGV screenshot of H3K27ac in late cerebellum.*

It is difficult to determine which genes an enhancer acts upon, as proximity is a fallible guide: intergenic enhancers do not necessarily regulate the nearest gene, and enhancers located within the intron of one gene can regulate an entirely different one. Methods like Hi-ChIP or PLAC-seq are required to understand which enhancers regulate which gene. We have defined active enhancers by their chromatin state alone. While this approach has limitations and probably does not reflect some of the complexity of gene regulation, it nevertheless remains a standard method in the field. In **Figure S9D** of the revised manuscript, we show just a single cluster of putative active enhancers near *Kcnk2*. H2AK119ub increases across each of these sites as the cerebellum matures, but H3K27ac changes in different ways (rising, falling or staying the same). The abundance of the *Kcnk2* transcript declines over time, but it is still expressed at low levels in the mature cerebellum (as shown in **Fig S9D**). Does this mean that H2AK119ub-mediated repression overpowers H3K27ac activation? We do not really know the answer, as there are probably additional histone modifications that are also changing. Therefore we have avoided speculating too much in these instances.

*4-Although it does not seem to be a correlation between K27me1 and RNA abundance in late cerebellum. Can authors test the early and late expression levels of the genes that gain K27me1 across development vs the ones that lose K27me1? (top 10% and bettom 10% in Fig 9D).*

We now include heatmaps of RNA data showing transcript abundance within the different deciles of K27me1. We also include heatmaps of H3K37me3 data, to show that sites that harbor K27me1 in the early cerebellum do not in general become subsequently trimethylated. There is not much change early versus late for the RNA. This observation supports our conjecture that changes in K27me1 likely play a greater role transcriptional memory .

*5-It is unclear to me why the number of peaks for each mark is different in the genome-wide aggregate heatmaps and in the volcano and log2-log2 plots in Fig 2-4. For instance, why are there 36,142 H3K27me3 peak summits in early cerebellum in Fig 2B and 12452 H3K27me3 peak regions in early cerebellum in Fig 2C,D? Maybe worth adding a note in the figure legend: i.e. note that number of peaks differe between Fig2B and C,D due to the use of different tools for peak definition (MACS2 narrow vs SICER).*

The differences the reviewer notes are due to the fact that each peak region usually harbors multiple summits. We use different peak calling algorithms for these two purposes because SEACR calls only one peak summit per region. We have included a comment on this difference in the legend for **Figure 3C**.

*6-It is unclear what is the difference between Fig 4B and Fig S6A and Fig 4C and S6B. Do perhaps the aggregated heatmaps in Fig S6A, B correspond to the marks in late cerebellum (instead of early, as indicated)?*

Thank you for catching this error: this plot was inadvertently mislabeled and has been corrected.

*7-Bar graphs in Fig S7 seem accidentally displaced downwards.*

Thank you for catching this as well; this error has been corrected.

*8-Can authors show pie chart of fractions of H3K27me1 peaks localized in different genome regions? This would support the statement in lines 429-431*

Excellent suggestion. We performed this analysis in **Figure 9B**, and, satisfyingly, the result is exactly as predicted.

*9-There are few typos need to be corrected:*

*-Line162, ‘…early cerebellar confirmed…’ should be ‘…. early cerebellum confimed…’.*

*-Line 183, one of the two H3K27ac should be H3K9ac.*

*-The panels described in Fig 3 legend start from (E), should start from (A).*

We very much appreciate this reviewer's sharp eye! We have made these corrections.

*10-Details for computational analysis in the methods, are sometimes limited. Depositing the code generated for many of the analysis in GitHub, and referencing it in the final version of the manuscript would be convenient for others, like myself.*

As recommended, we have deposited our code on GitHub under FergusonLab with a link provided. Our hope is that others in the community will use these tools.

**Reviewer 2**

*Srinivasan and Parmar et al claim that H2AK119ub spreads to euchromatin, not just facultative heterochromatin, independently of PRC2 during the development of the cerebellum from the early to late stages. Indeed, as shown in Figure 2C,D, the developmental shift from early to late stages shows that H2AK119ub and H3K27me3 do not necessarily colocalize. This can be interpreted to mean that PRC1 and PRC2 begin to exert independent control. From this perspective, the question of which chromatin regions H2AK119ub extends its distribution to is highly interesting for exploring new functions of PRC1.*

*The most important observation in this study is shown in Figure 1E: at both Early and Late stages, there are two patterns of uH2A – one that colocalizes with H3K27me3 and another that colocalizes with H3K27ac. These two types of uH2A modifications are likely driven by completely separate mechanisms. Regarding the regions where uH2A colocalizes with H3K27ac, there are two points of novelty. First, it is highly probable that this represents a regulatory mechanism independent of the repressive mechanism that works in concert with PRC2, showing dynamics that cannot be explained solely by the traditional model of "H2AK119ub recruiting H3K27me3"(Rob, Mol Cell, 2019). The second point is that these regions are observed much more prominently in the Late stage of cerebellar development, suggesting that this PRC2-independent regulatory region propagates widely towards the later stages of development. The authors focus on this second point of novelty, demonstrating its localization distribution and functional significance using cerebellar development as a model. This is a important novel perspective in chromatin research.*

*However, because the authors analyze the peaks of uH2A in the early and late stages independently, it is difficult for me to imagine how uH2A and other histone modifications change coordinately or antagonistically over the developmental time course. This contributes to a very unclear structure. Furthermore, most of the data presented lacks transparency in its overall quantification. For example, while summarizing the trends and correlations of each histone modification in early/late stages with correlation coefficients, as shown in Figure 5, is very clear and novel in terms of data presentation and analysis methods, it is difficult to readily accept without showing visualized heatmaps or concrete track views for individual gene loci and regions. To allow reviewers to better understand how the data was compiled and led to specific conclusions, adding figures that visualize the data closer to its raw form is recommended. This would improve the study's transparency and enhance the reliability of the results.*

*From the above points, the authors' findings are highly novel and interesting for the field of Polycomb and chromatin research, but they are difficult to accept in their current state. Adding the following data and experiments would make the manuscript more acceptable.*

We are immensely grateful for this reviewer’s feedback, which has enabled us to clarify our framework as well as the interpretation of our findings. We have also taken their recommendations that we display data in a way that more closely resembles its raw form. We trust that the reviewer will find our revised manuscript more compelling.

*Major Comments*

*•The authors conclude that uH2A distributes to euchromatin in the later stages, based on CUT&RUN data for H3K27me3 and euchromatin histone modification marks. Figure 2 examines the movement of uH2A based on H3K27me3 peak calls, Figure 3 based on H3K4me3, and Figure 4 based on H3K27ac. However, the clearest way to present this would be to examine the results based on uH2A peak calls. The authors should analyze how the regions that colocalize with H3K27me3 change from Early to Late after calling uH2A peaks, and then re-analyze which histone modification patterns the uH2A-unique peaks coincide with. If the results are consistent with Figures 2-4, then Figures 2-4 could be moved to supplementary data.*

This was a very astute recommendation that led to our restructuring the entire results section. By making H2AK119ub peak calls the 'baseline' and then analyzing which marks coincide with these peaks, we found that all of the other modifications behave differently in these distinct chromatin contexts. Sites of coordinated PRC1-PRC2 activity are subject to different neurodevelopmental regulation than sites of just PRC1 activity, with the latter class exhibiting greater overall dynamism across modifications.

*•Regarding the four patterns of track views shown in Figure 1E, the authors should create a heatmap to show their genome-wide generality and indicate the number of regions for each pattern. Furthermore, for these four patterns, the authors should investigate whether the H3K27me3/H3K27ac levels in the Late stage all change similarly, or if they show diverse patterns of change. The results should be checked to confirm they are not inconsistent with Figures 6C and 6G.*

After reflection, we decided to remove the previous Figure 6 entirely and now show how euchromatin- and heterochromatin-associated modifications change in relation to H2AK119ub not only through locus-level data visualization but also through more Log2-Log2 plots and heatmaps centered around sites with increased, decreased, or unchanged H2AK119ub. These visualizations are easier to interpret than the hierarchical clustering in the old Figure 6 and clarify changes in chromatin state between early and late stages.

*•Observing data like Figures 1F,G, regions that initially had H3K4me3 or H3K9/27ac deposition and showed RNA transcription in the early phase appear to have increased H2AK119ub/H3K27me3 and suppressed transcription in the late stage. This appears to be simply repression by PRC1/2 in the late stage, rather than H2AK119ub spreading into Euchromatin. It is unclear where the novelty lies in this case. Are these loci not representative of the authors' main claim? It is more important to show the CUT&RUN data for each histone modification shown in Figure 1E for both Early and Late stages.*

At many loci, repression is accompanied by loss of activating modifications in regions of both PRC1 and PRC2 activity. As we show in the new **Figure 1**, however, these same changes (repression, loss of activating modifications) can occur in loci that undergo developmental ubiquitination in the absence of concurrent H3K27me3 deposition. Our efforts to separately consider changes in chromatin environment within H2AK119ub-only sites versus sites harboring H3K27me3 in combination with H2AK119ub addresses this question in the most comprehensive way possible with our data.

*•As shown in Figure 6G, uH2A increases in all clusters regardless of changes in other histone modifications. This could be interpreted as simply a result of clustering based on the uH2A peak calls in the Late stage. Indeed, Figure 6B shows regions where uH2A decreases. This issue arises because independent populations are analyzed, which can cause confusion for the reader. Therefore, the authors should show how the number and movement of uH2A peaks change from early to late using Venn diagrams or similar based on the peak call results for both Early and Late stages. Based on this, it would be clearer to divide regions into those where uH2A does not change from early to late, those where it decreases, and those where it increases, and present this as a heatmap. If, among these changes, there are regions where uH2A changes not only coordinately with H3K27me3 but also with euchromatin marks, or if the binding of uH2A in such diverse chromatin regions increases in later development, the authors' claim that uH2A propagates to euchromatin regions during cerebellar development would be convincing to everyone.*

This insightful comment led to an overall clarification of the manuscript’s structure and conclusions. We have used DiffBind to identify sites in which H2AK119ub significantly rises, falls or stays the same. This analysis resulted in an entirely new **Figure 2**. We go on to examine H3K4me3, H3K27ac, H3K9ac and H3K27me3 within these classes of loci using aggregate heatmaps. These analyses convincingly show that activating modifications move in concert with uH2A, whereas K27me3 does not. This underscores one of our main findings, namely that PRC1 and PRC2 begin to operate independently at many sites as the brain matures.

In the new **Figure 6** we show the degree of overlap between early and late peak regions for all of the modifications, including uH2A. This analysis showed that uH2A peak regions were less similar to one another between the early and late cerebellum than was the case for the other modifications, whose peak regions exhibited a greater degree of overlap between the timepoints. This corroborates our claim that uH2A is more dynamic.

*•Is the chromatin remodeling of uH2A and H3K27me1 entirely restricted to CGI regions? Is it possible to explain the PRC2-dependent and independent forms of uH2A by differences in the presence, density, or length of CGIs?*

This is an interesting hypothesis. PRC1 activity is not restricted to CGI regions, and most enhancers are not associated with CGIs. It is entirely possible that PRC1/PRC2 collaborate within CGIs, whereas PRC1 operates independently of PRC2 at non-CGI sites. However, analyzing the density of CGIs in comparison to the different classes of regions where H2AK119ub appears and changes during neurodevelopment is a sophisticated analysis that should be explored in a future study, where we can do justice to the complexity of this question.

*Minor Comments*

*•In Figure 1E, including CUT&RUN data for H3K27ac and H3K27me3 in the Late stage as well, and showing how the chromatin state changes in these regions between Early and Late stages before showing the uH2A changes would be clearer.*

We have changed the order in which these data are presented so that examples of how uH2A can change within heterochromatin appear within the figure describing how uH2A changes in heterochromatin overall genome-wide (**Figure 3**). Likewise, we have included examples of how uH2A can change within euchromatin in the figure that describes this phenomenon more generally through genome-wide analyses (**Figure 5**). Thanks in large part to the addition of analyses recommended by this reviewer, this revised presentation does a much better job of describing the changes in H3K27me3 and H3K27ac within sites where H2AK119ub increases, decreases or stays the same. In this way, we have addressed the general case. We have also done a more thorough job of simultaneously comparing changes in H2AK119ub to changes in H3K4me3, H3K27ac and H3K27me3, which addresses the genome-wide trends much better than showing a few example loci. These example loci are presented at the outset of these figures simply to show that there is no single pattern, and only genome-wide analyses can reveal the bigger picture.

*•In figures like Figure 8C and Figure 9B, the correlation coefficient results should be shown in the figures.*

We have modified this figure panel considerably. To begin with, we have now excluded loci harboring H3K27me3 at their promoters: in this way, we consider only the expression of protein-coding genes in comparison to H3K27me1 abundance, without the confounding effect of including loci that are silenced via H3K27me3, which will also harbor little H3K27me1. However, in the resubmitted version we have chosen not to show the correlation coefficients in these plots. These values turn out to be somewhat confusing because most points lie near the origin, having low RNA expression and low H3K27me1. Therefore the correlation coefficient ends up being slightly positive. In our opinion, this obscures the true interpretation of this figure, which is instead that highly expressed loci tend to have low amounts of H3K27me1.

*•Combining Figures 8A and 8B to show track views of K27me1, K27me3, K4me3, and RNA for both Early and Late stages would be clearer. Also, at this time, is uH2A increasing?*

We have now included tracks of RNAseq data in **Figure 8A**, which is in clear agreement with the heterochromatin-euchromatin partitioning, as RNA signal is present only in euchromatic zones. But the point of this figure is not to compare early and late K27me1, but rather to compare the distribution of K27me3, K4me3 and RNA to K27me1. Like the rest of the genome, K27me1 undergoes a consolidation process within genes in this region over time. We looked at uH2A in this region, and it turns out to not be very dynamic. In our view, including data from different timepoints, along with uH2A data, would muddle the main message in this figure. We consider the dynamism of K27me1 with the appropriate nuance and thoroughness in Figure 9.

*•In Figure 8 and beyond, where early H3K27me1 is broadly and uniformly distributed within and outside genes, and later becomes significantly enriched within specific gene bodies, what is happening with uH2A? If both H3K27me1 and uH2A are increasing, could it be interpreted that one reason for the decrease in H3K27me3 colocalization with uH2A is that uH2A is instead associated with H3K27me1?*

Excellent question. We checked and there is simply little ubiquitination at the genes where K27me1 becomes consolidated. K27me1 is associated with gene bodies, while uH2A is associated with cis-regulatory elements, so they do not appear to colocalize.

*•Are the data regarding the distribution of uH2A in the first half of the paper and the data on H3K27me1 being concentrated in specific genes discussed as separate mechanisms?*

Yes, they are largely being discussed separately because their genomic distributions differ so much. If there is a connection between PRC1-dependent uH2A and PRC2-dependent K27me1, possibly through a DNA element such as CGIs or polycomb-response elements, it will be a complicated question that will have to be explored in subsequent studies.

**Reviewer 3**

*This is an interesting manuscript reporting compelling chromatin data that reveal new ways of thinking about polycomb complex functions in neuronal differentiation. The authors use the relative homogeneity of cell types in the mouse cerebellum to study how changes in distinct histone modifications representing the function of different PRC components change over the course of cellular differentiation. They are especially interested in the H2AK119ub mark deposited by the PRC1 complex, which has traditionally been associated with gene repression. Here as they characterize the distribution of this modification across chromatin in immature (P12) versus mature (3 months) cerebellum compared with other well characterized histone marks of chromatin compartments. They find that the distribution of H2AK119ub significantly colocalizes with the PRC2 induced mark H3K27me3 in P12 brain, consistent with a role in facultative heterochromatin. However they also find H2AK119ub at some active enhancers (H3K27ac+/H3K4me1+) suggesting that it might have non-canonical functions in euchromatin. By 3 months the distribution of the two marks diverges with H2AK119ub levels rising at regulatory elements in euchromatin and falling in facultative heterochromatin. These data enrich understanding of PRC1 functions that are independent of PRC2. The data are presented and evaluated in rigorous fashion – this was one of the best descriptions of bioinformatics choices for pipelines and normalization strategies that I have seen. I have only minor concerns.*

We truly appreciate the reviewer's enthusiasm for this study and their appreciation of our bioinformatics analyses (or at least their description!). We have made our code accessible to the community by posting it on the FergusonLab GitHub.

*1) The authors do quite a bit of filtering for peaks that have a histone mark with known meaning (such as H3K27me3 for facultative heterochromatin or H3K4me1 for poised enhancers) and then assess H2AK119ub with respect to these other chromatin domains. However it would be useful if they would also just show an overall MA plot of H2AK119ub changes between the two time points. Summary statistics for the genome distribution of this mark (exon/intron/etc) as well as the overall correlation with changing gene expression would be useful as well. These can kind of be derived from other data presented, but the manuscript would benefit from starting with data on this mark alone before proceeding to the comparisons.*

This was excellent advice, which we acted upon by performing DiffBind analysis of H2AK119ub data from early in comparison to late cerebellum in the new **Figure 2**. This allowed us to identify loci that underwent no change versus an increase or decrease in H2AK119ub, which we then used to examine other histone modifications in relation to these classes of H2AK119ub-positive loci. We also annotated H2AK119ub-positive loci in each of these categories, discovering that most sites were found in introns, intergenic or promoter regions, greatly strengthening our assertion that H2AK119ub localizes primarily to cis-regulatory elements.

*2) Line 179 “there was no consistent pattern of enrichment or depletion…” That is not really true that there is no pattern, there is just more than 1 so perhaps saying no singular pattern rather than consistent?*

We agree completely and have changed the language accordingly.

*3) There are multiple H2A variant histones – does the composition of histone variants change over time in these neurons and is there any difference in the regulation of different variants by ubiquitination at K119? If it is relevant, a comment could be added to the discussion.*

This is quite an interesting idea, but addressing it is outside the scope of this already-lengthy paper. In another project in the lab, we have generated neuron-specific mutants in Bap1, the H2AK119ub deubiquitinase. These mutants have a very severe neurodevelopmental phenotype, with the H2A variant H2A.z markedly perturbed. So even though we do not address this topic in this study, we are currently studying the interaction between the regulators of H2AK119ub and histone variants. We do now mention in the discussion that H2A variants should also be considered in future studies of this kind.

*4) Atoh1 is an odd choice to show as a bivalent gene at P12. This gene is expressed much earlier in cerebellar development and has largely turned off by P12, whereas bivalent genes are usually discussed in the context of genes poised for activation. One possible concern is that P12 could actually be a mixture of developmental cell states. Depending on the strain, granule neuron progenitors (GNPs) can still be present in the second postnatal week, along with a mixture of newborn and maturing CGNs. In that case the apparent bivalency might instead reflect H3K4me3 at this promoter in GNPs and H3K27me3 at the promoter in CGNs rather than true bivalency in the same cells.*

This is an important point. We have eliminated this figure panel and done a more thorough job of analyzing the interaction between bivalent promoters and H2AK119ub, including effects on transcription.

*5) Given that many sites of H3K4me3 and H3K27me3 do not change between P12 and 3 months, it would be useful in some of the figures to subset dynamic versus static peaks to see how these compare against the changing H2AK119ub distributions.*

We have taken a slightly different approach to address the spirit of the comment by including several more log2-log2 plots to compare changes in H2AK119ub to changes in H3K27me3, K4me3 and K27ac. These new analyses have been highly revealing. K27me3 and H2Aub are largely independent of each other, but K4me3 and H2Aub tended to move in the same direction, indicating that gain of H2Aub at active promoters is typically accompanied by increasing amounts of K4me3. These effects were present whether we considered K4me3 peak regions in the early or late cerebellum.

Interestingly, however, the relation between H2Aub and H3K27ac changed, whether we considered H3K27ac peak regions in the early or the late cerebellum. Within K27ac peak regions from the *early* cerebellum, increases in H2Aub tended to be accompanied by a decline in H3K27ac. In contrast, within K27ac peak regions identified in the *late* cerebellum, H2Aub and K27ac both tended to increase. This indicates that H2Aub regulates active enhancers differently in the mature versus the developing cerebellum, a crucial new insight of broad relevance to gene regulation during neurodevelopment.

*6) The CpG islands are called “promoter-associated” but S5A clearly shows one that is intragenic. Did the authors use all CpG islands or only those in certain genetic regions?*

We used all CpG islands for these analysis. The two CpG islands in the *Bcat1* locus are associated with different transcription start sites. The downstream CpG island on the left side of this figure panel is associated with the promoter that is used in neurons, as the RNAseq signal begins there. There is no RNA signal in between the two CpG islands that are shown.

*7) Figure 4G is not mentioned in the results.*

Thanks for catching this inadvertent omission. We have included a description of this panel in the revised manuscript.

*8) Line 303 the phrase “rather closely mimicked” is difficult to interpret. The figure shows overlap at some sites, but is there a way to quantify the similarity? This may be in Figure S7 – this was difficult to tell because the formatting of S7 is off in the downloaded version.*

We have eliminated this confusing language and corrected the mentioned plot (**figure S11** in the revised ms).

*9) I am confused about line 311 where the authors talk about PRC1 being redistributed toward enhancers at the neural progenitor stage. This comes after the authors conclude that between P12 and 3 months that H2AK119ub is gained in euchromatin in CGNs. However if there are neural progenitors in cerebellum they are certainly there at P12 not 3 months. I think the authors are probably suggesting that in ref 43 the NPC stage is the more mature stage compared with something else, but this was unclear.*

We agree that this was confusing and have eliminated this statement and the reference that accompanied it.

*10) Line 362 the authors cannot really say PRC1 shifts its “regulation” – they show it shifts its “distribution” via the distribution of the mark.*

We agree and have modified the language accordingly.

*11) Line 364 the authors cannot say that H2K119ub and H3K27me3 “operate” independently at “most” loci in the cerebellum. They did not show this. They show that places where H2K119ub increases are not places that H3K27me3increases. They do not prove a lack of overlap. And definitely they cannot say anything about function.*

We have added a great deal of data related to this point. In particular, we analyze sites where H2Aub and K27me3 peak regions overlap compared to sites marked by H2Aub alone. The number of loci in the latter category greatly outnumber those in the former. Furthermore, H2Aub and H3K27me3 move in opposite directions over the course of neurodevelopment within loci that harbor H2Aub alone. This indicates that PRC1 and PRC2 become independent of each other as the cerebellum matures (see also Reviewer 2’s comments and interest in this question).

*12) Line 364 I do not see data that support the statement that “H2AK119ub localizes mainly to enhancer regions”.*

In Figure S11, most peak regions for H2Aub overlap with H3K4me1, which is associated with enhancer activity. This is the origin of our assertion. Furthermore, analyzing the distribution of H2Aub peak regions in Figure 1 demonstrated that most sites were in intergenic or intronic regions (i.e., associated with enhancer function) far more than H2Aub localized to promoters.

*13) Dividing versus non-dividing cells have fundamental differences in the ways the regulate distribution of their chromatin marks. For the cerebellum vs liver vs kidney comparison, what percentage of the kidney and liver cells are mitotic? Did the authors consider comparing their P12 cerebellar data to P4 or P7 cerebellum (or isolated granule neuron progenitors) where there would be more mitotic cells but all in the CGN lineage?*

There are not very many dividing cells in healthy nonneural organs; most references describe 10% as the upper limit. The vast majority of cells in these organs are quiescent. In the cerebellum we wanted to avoid the contribution of mitotic neural progenitors for the very reasons outlined in the reviewer’s comments. The choice of the appropriate early time point for the cerebellum was a balancing act between representation of immature neurons and avoiding the variability that comes when mitotic cells are present. Wait too long and many neurons may be too far along in the differentiation process, with the oldest neurons having exited the cell cycle 10 days prior; go too early and the variability is too high due to waves of proliferation and the abundance of mitotic cells. The P12 timepoint is commonly chosen to balance these competing interests in bulk assays that use the cerebellum as input. Comparing earlier timepoints would be interesting, but would have to be explored in future studies.

*14) Are the GO terms in Fig 7G-H for the closest gene or did the authors consider only promoter/ intragenic peaks that were easily mapped to a gene?*

We mapped to the nearest gene, regardless of whether loci were located within promoters, intragenic or intergenic regions.

*15) Regarding the plasticity of H3K27me3 distributions in developing CGNs, and the role of EZH1 and EZH2, the authors would do well to contextualize their data with other studies. A prior study already reported the regulation of H3K27me3 at multiple stages of CGN differentiation (PMID: 37092728) and that study should be cited here. Both that study and Ref 71 in the reference list used pharmacological inhibition of EZH1 or EZH2 to ask about the roles of these two enzymes in H3K27me3 distributions in CGNs and found roles for both. Finally with respect to the question of deposition versus maintenance of H3K27me3, the authors may want to check out PMID: 27526204, which showed that knockout of both Ezh1 and Ezh2 in adult brain is required to lead to loss of H3K27me3, demonstrating a role for both enzymes in mature neurons.*

In the revised manuscript we have cited the paper from Anne West’s lab (*eLife* 2023, the first paper mentioned above) to demonstrate what is known about the role of PRC2 in heterochromatin during cerebellar maturation. It was an oversight not to include this study, so we are thankful for the reviewer bringing this to our attention. We have also cited the paper from Anne Schaefer’s group (*Nat Neurosci* 2016, the other PMID given above) in two locations – in the Introduction to demonstrate the importance of PRC2 activity for preventing neurodegeneration, and in the Results to establish that both EZH1 and EZH2 are necessary for H3K27me3.

While these studies examined H3K27me3 in the context of neurodevelopment, none of them examined H3K27me1, let alone its dynamic behavior within euchromatin during neurodevelopment, which is a main focus of our study. Data from not just our lab but also the van Zundert group (PMID 23932971, Henriquez et al., *Mol Cell Neurosci* 2013) show that EZH2 is more highly expressed during early development, whereas EZH1 is more stably expressed in mature neurons. It is no coincidence that continued expression of EZH1, which readily generates H3K27me1 but not H3K27me3, is critical to the remodeling of the distribution of H3K27me1, perhaps in a coordinated way with the reduced expression of EZH2.

*16) Line 389 “(Figure 7A-5C)” Presumably this is a typo.*

Yes, indeed: thank you for catching this, which we have now corrected.

*17) Figure S6J I am not sure that the shared fraction can be described as a “sizable minority” when for H2AK119ub it appears to be about 45% in most of the comparisons. It seems like the authors should also show “shared” peaks among the three tissue types as well as combinations of “unique” peaks between two pairs to match the “cell-type specific” statement.*

If 45% is *not* a sizable minority—and it certainly cannot be ‘a miniscule majority’!—then we interpret this comment as suggesting we use “nearly half” to describe the shared fraction. Fair enough. The main comparison is between the cerebellum and the non-neural organs. In the revised manuscript we list the number of unique peak regions in **Figure 7**, as well as the number of peak regions that were found across all three organs.

*18) Line 849 “To Analysis”? Presumably this is a typo.*

Alas, yes. We have corrected this error.
